# Outcome of Atrial Fibrillation Ablation in Cancer Patients: A Review

**DOI:** 10.7759/cureus.47818

**Published:** 2023-10-27

**Authors:** Nava R Sharma, Arjun Basnet, Saral Lamichhane, Sajog Kansakar, Armando Seitillari, Marlon E Rivera Boadla, Sudarshan Gautam, Prabal KC, Kripa Tiwari, Aniruddha Singh, Sijan Basnet, Bikal Lamichhane, Madalasa Pokhrel

**Affiliations:** 1 Medicine, Manipal College of Medical Sciences, Pokhara, NPL; 2 Cardiology, Tower Health Medical Group, West Reading, USA; 3 Internal Medicine, Gandaki Medical College, Pokhara, NPL; 4 Internal Medicine, Manipal College of Medical Sciences, Pokhara, NPL; 5 Internal Medicine, Maimonides Medical Center, Brooklyn, USA; 6 Internal Medicine, Rasuwa District Hospital, Kathmandu, NPL; 7 Internal Medicine, The Reading Hospital and Medical Center, Wyomissing, USA; 8 Internal Medicine, Guthrie Robert Packer Hospital, Sayre, USA; 9 Internal Medicine, Montefiore Medical Center, New Rochelle, USA

**Keywords:** atrial fibrillation and cancer, cardiac catheter ablation, radiofrequency catheter ablation, cancer patients, atrial fibrillation (af)

## Abstract

Atrial fibrillation (AF), a cardiac arrhythmia, exhibits a heightened prevalence among individuals diagnosed with cancer, notably prominent in cases of lung and gastrointestinal malignancies. Robust evidence from extensive studies underscores this association, emphasizing its clinical significance. However, the precise mechanistic underpinnings and specific risk factors linking cancer and AF remain a subject of incomplete understanding. Notably, the prevalence of AF in cancer patients substantially exceeds that in non-cancer counterparts, prompting further exploration of the underlying pathophysiological processes.

This review aims to address the existing knowledge void regarding AF management in cancer patients, with a specific focus on the potential role of ablation procedures. While catheter and surgical ablation techniques have been thoroughly investigated and validated as effective treatments within non-cancer populations, their applicability and outcomes in cancer patients have remained inadequately explored.

The principal objective of this exhaustive review is to bridge this research gap by conducting a meticulous examination of the feasibility, safety, and effectiveness of ablation interventions for AF in the context of cancer patients. By amalgamating existing evidence and pinpointing critical areas necessitating additional investigation, this review endeavors to provide invaluable insights into AF management in cancer patients, with the ultimate goal of enhancing their clinical care and optimizing outcomes.

## Introduction and background

Atrial fibrillation (AF) is a cardiac arrhythmia that has been observed to occur at a higher rate in individuals diagnosed with cancer compared to the general population [1]. Notably, lung and gastrointestinal cancers have been associated with a significant increase in AF incidence [2]. Large-scale studies involving over 300,000 cancer patients have confirmed this association across various cancer types, but the underlying mechanisms and specific risk factors remain poorly understood [3]. Another study found that the prevalence of AF in cancer patients was 9.77%, whereas, in the non-cancer population, it was 1.19% [4]. The exact mechanisms underlying the induction of AF in cancer patients are not fully understood.

Further research is needed to fully understand these mechanisms' specific contributions and develop targeted interventions for AF in cancer patients. Despite recognizing AF as a common comorbidity in cancer patients, there is a notable research gap regarding the role of ablation procedures in managing AF within this population. The efficacy and safety of ablation techniques, such as a catheter or surgical ablation, have been extensively studied in non-cancer populations. Still, their application and outcomes in cancer patients have not been thoroughly explored. This review article aims to bridge this research gap by providing a comprehensive analysis of the potential role of ablation in treating AF in cancer patients.

## Review

Methodology

A comprehensive search of relevant databases, including PubMed, Embase, and Google Scholar, was performed using a combination of keywords and Medical Subject Heading (MeSH) terms, including "atrial fibrillation," "cancer," "carcinoma," "malignancies," "tumors," "catheter ablation," and "ablation" using the Boolean operators. The search was limited to articles published in English from 2000 to the present; the final examination was conducted on August 3, 2023. The papers were screened for relevance by title and abstract, and duplicates were removed. The full texts of potentially relevant articles were reviewed, and the key findings were summarized in table form. A narrative discussion also included the relevant results, limitations, and gaps in the current evidence.

Atrial fibrillation in cancer patients: pathophysiology

Several factors strongly link AF in cancer patients. The general population's established risk factors for AF include chronic kidney disease, hypertension, hyperthyroidism, heart failure, hyperlipidemia, alcohol consumption, smoking, and advanced age [5-7]. These risk factors can also affect AF in cancer patients. The development of AF is also influenced by several comorbidities commonly found in cancer patients, including anemia and metabolic disorders. Specific risk factors for AF in cancer patients are associated with physical or emotional stress, such as pain causing sympathetic system activation.

**Figure 1:**
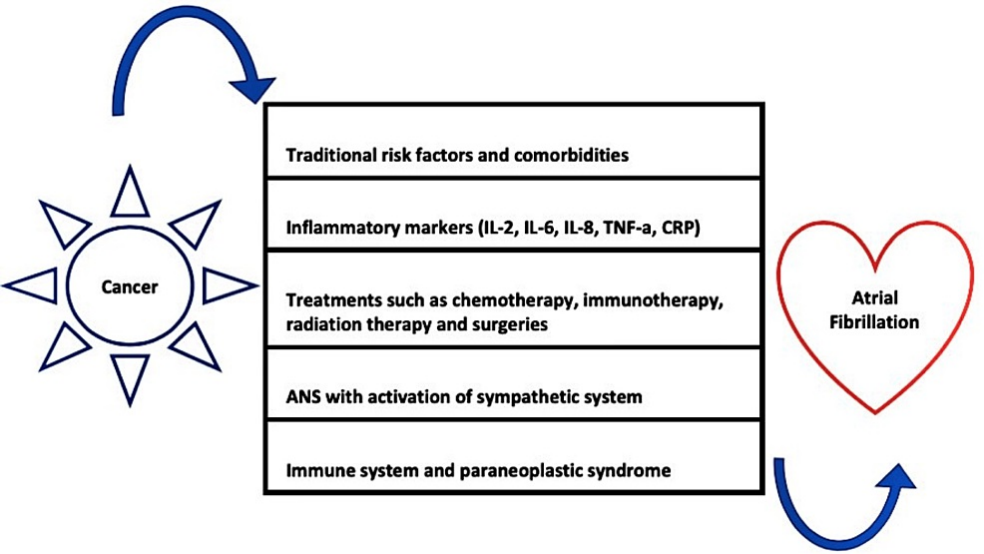
Schematic diagram of underlying mechanistic processes involved in the pathophysiology of AF in cancer patients. AF: atrial fibrillation; IL: Interleukin; TNF-a: tumor necrosis factor-alpha; CRP: C-reactive protein; ANS: autonomic nervous system. Image credit: Dr. Arjun Basnet.

The exact mechanisms underlying the induction of AF in cancer patients are not fully understood. The causation of AF in cancer patients involves complex and multifactorial mechanisms of chronic inflammation, oxidative stress, autonomic nervous system dysregulation, cardiac structural abnormalities from cancer therapies, electrolyte imbalances, and the prothrombotic state associated with cancer [8,9]. Also, chemotherapeutic drugs such as cisplatin have shown an incidence of AF ranging from 15% to 32%, while anthracyclines have been linked to AF in approximately 10% of cases [10]. The interplay of these factors disrupts regular atrial electrical activity and increases the vulnerability of the atria to arrhythmias [8]. Moreover, cancers often induce a state of high inflammatory stress, and various cancer treatments like radiation therapies, chemotherapies, and immunotherapies, are cardiotoxic, significantly increasing the risk of AF onset [6,8,11]. Cancer elevates inflammatory markers, which have been implicated in left atrial remodeling and fibrosis. Interleukin 2 (IL-2), IL-6, IL-8, tumor necrosis factor-alpha (TNF alpha), and C-reactive protein (CRP) are among the inflammatory markers believed to be associated with an increased risk of arrhythmia as well as commonly affected by various malignancies [12,13]. Another connection between cancer and AF involves the immune system. Certain cancers can trigger autoimmune paraneoplastic syndromes, leading to structural alterations in the left atrium and facilitating AF [14]. A comprehensive summary of the various risk factors in cancer patients that influence the development of AF has been depicted in Figure 1.

Challenges associated with the management of atrial fibrillation in cancer patients

AF in cancer presents a unique clinical challenge. Several chemotherapeutic agents have been associated with AF. Drugs like vinca alkaloids, cisplatin, doxorubicin, and azathioprine, among others, cause AF and potentially trigger its recurrence [7]. Direct cardiotoxicity from these agents increases the risk of developing AF. Moreover, the rising utilization of monoclonal antibodies and targeted therapies has seen a parallel increase in cardiovascular morbidities, including AF.

Active malignancy amplifies the thromboembolic risk in patients with AF due to its hypercoagulable state, creating a challenge in anticoagulation [15]. Thrombocytopenia due to chemotherapy-induced bone marrow suppression can further complicate this situation. Current risk prediction models like CHA2DS2-VASc, aimed at assessing thromboembolic risk, and HAS-BLED, assessing bleeding risk, lack robust validation in the oncologic demographic [16]. Anticoagulation decisions warrant a highly individualized approach, factoring in unique risks, possible drug interactions, and patient preferences.

Many chemotherapeutic agents possess nephrotoxic properties, leading to impaired renal function. This impairment can affect the renal clearance of antiarrhythmic drugs (AADs). Consequently, dose modifications, grounded on the glomerular filtration rate (GFR), become imperative for safe and effective therapy [17]. Hence, the interplay between AF and cancer presents intricate clinical challenges that demand careful consideration.

Many of the guiding principles for considering rate or rhythm control (AAD, catheter ablation (CA), surgical ablation, and electrical cardioversion) are similar for cancer patients as they are for the general population [18,19]. However, monitoring for drug interactions of rate control agents, AAD, and anticoagulants with chemotherapeutic agents may become necessary [19]. Additionally, it is essential to note that AAD therapy, CA, and electrical cardioversion have not been studied adequately in cancer patients. There are no trials assessing the safety and efficacy of CA in cancer patients. Landmark clinical trials for AF CA, such as CABANA, CASTLE-AF, and EAST-AFDNET-4, focused on cardiovascular outcomes with no specific mention or inclusion of cancer patients [20-22].

National Inpatient Sample (NIS) database studies indicate that comorbid cancer in patients with AF is associated with increased all-cause in-hospital mortality. Active cancer was associated with increased in-hospital mortality in AF patients who underwent CA [23-25]. However, these studies should be interpreted carefully due to the lack of robust statistical methods and inherent study design limitations, such as sampling bias, lack of longitudinal data, coding bias, and lack of outpatient or follow-up data.

Most of the evidence for the safety and efficacy of CA for AF in patients with active malignancy or a history of malignancy comes from observational studies. The most extensive retrospective cohort study by Ganatra et al. evaluated outcomes of AF ablation in patients with a history of cancer [26]. The study included 502 patients, with 251 having a history of cancer, of which 18.3% had active cancer, and the rest were in remission. The results showed that freedom from AF at 12 months and the need for repeat ablation did not differ significantly between the study and control groups. Similar rates of complications were seen. Similar results were seen in a prospective study, where complications and arrhythmia-free survival rates at 12 months were similar in patients with a history of cancer (8/70 with active cancer) [27]. A study by Wang et al. included patients with active cancer (n = 30) and patients without cancer (n = 60). It showed no significant difference in the recurrence of AF after CA in patients at one year [28]. However, in one study, cancer survivors were seen to experience an increased rate of clinically significant bleeding after radiofrequency CA (OR = 3.60; 95% CI = 1.02-12.73) [29]. There was no significant or fatal bleeding reported.

Data regarding the risk of complications and treatment failure rate among patients with active cancer undergoing CA for AF are limited. Evidence from observational studies shows no difference in the efficacy of CA between the two groups with similar recurrence rates. However, findings regarding procedural bleeding risk were mixed. It is difficult to draw definite conclusions due to insufficient high-quality evidence from clinical trials. Only a few observational studies with small sample sizes exist, with active cancer patients representing a small percentage (Table 1).

**Table 1:** Safety and efficacy of AF ablation in cancer patients. AF: atrial fibrillation; CA: catheter ablation; NA: no information available; AAD: antiarrhythmic drug; TIA: transient ischemic attack; MI: myocardial infarction; aOR: adjusted odds ratio; DCCV: direct current cardioversion; HCUP: Healthcare Cost and Utilization Project; NIS: National Inpatient Sample.

Reference	Study design	Target population	Sample size	Number of participants with a history of cancer	Number of participants with active cancer	Follow-up period	Results or outcome	Limitations
Ganatra et al. (2023) [26]	Retrospective cohort study	Patients with a history of cancer within five years before or those with exposure to anthracyclines and/or thoracic radiation at any time before the index ablation.	502	251	NA	12 months	Freedom from AF at 12 months with or without AAD did not differ between the study and control groups (83.3% vs. 72.5%, p = 0.28). The need for repeat ablation was similar between groups (20.7% vs. 27.5%, p = 0.29).	There was selection bias among the patients referred for CA. This study has a low representation of active cancer patients (18.3%) undergoing CA for AF. The types of cancer and cancer therapy included in the study were heterogeneous.
							Freedom from AF without AAD at 12 months was higher in the study group (50.6% vs. 35%, p < 0.001).	
							There was no statistical difference between groups within the first three months post-ablation, access and non-access site bleeding, pulmonary vein stenosis, stroke, and cardiac tamponade.	
Wu et al. (2022) [24]	United States Nationwide Readmission Database Case-Control Study	Patients with active cancer or a history of malignancy undergoing catheter ablation for atrial fibrillation compared with patients without cancer.	46,461	5133	800	Three years	Patient with active cancer has higher inpatient hospital mortality (1.63 vs. 0.31), procedural complication rate (10.44 vs. 6.43), and all-cause readmission rate (12.59 vs. 11.24).	NA
							Active cancer diagnosis but not a history of cancer was associated with increased hospital mortality (odds ratio: 2.72 (1.18-6.24), p = 0.018 vs. non-cancer).	
							Only a history of cancer was associated with a small increased risk of 30-day readmission following CA (hazard ratio: 1.19 (1.04-1.37), p = 0.009 vs. non-cancer).	
							There was no difference in procedural risk between cancer and non-cancer patients.	
Thotamgari et al. (2023) [25]	Study based on NIS database	Patients with cancer and atrial fibrillation undergoing catheter ablation.	57,730	1355	NA	Two years	Patients with cancer had significantly higher mortality (aOR = 1.67, 95% CI = 1.18-2.34, p = 0.003) and higher prevalence of acute MI (aOR = 1.53, 95% CI = 1.22-1.92, p < 0.001) but lower prevalence of stroke/TIA (aOR = 0.59, 95% CI = 0.48-0.72, p < 0.001) and cardiogenic shock (aOR = 0.15, 95% CI = 0.09-0.25, p < 0.001).	NA
							There was no significant difference in intracranial hemorrhage or cardiac arrest.	
Eitel et al. (2021) [27]	Prospective cohort study	Cancer survivors and active cancer patients with AF.	140	62	8	Cancer cohort: 606.7 ± 350.8 days. Cancer cohort: 670.4 ± 396.6 days	Arrhythmia-free survival after 12 months did not differ significantly in patients with and without a history of cancer (67.1 ± 5.8% vs. 77.8% ± 5.1%, p = 0.16).	There was a low number of patients with active cancer. There was a heterogeneous study population.
							Repeat ablation procedures were performed in 8 patients (11.4%) in the cancer group and 16 patients (22.9%) in the non-cancer group (p = 0.11).	
							The frequency of complications was similar between patients with and without a history of cancer (p = 0.11).	
Wang et al. (2021) [28]	Single-center retrospective study	Patients with malignant tumors and atrial fibrillation underwent radiofrequency ablation for the first time.	90	60	NA	328.7 ± 110.3 days	The atrial fibrillation recurrence rate was 20% in the tumor group compared to 28.3% in the control group(p > 0.05).	There was a lack of generalization, given that it is a single-center retrospective study. The study sample and event size were small.
							Kaplan-Meier survival analysis showed that suffering from a malignant tumor did not affect the recurrence rate of atrial fibrillation after radiofrequency ablation (p = 0.383).	
							Multivariate Cox regression analysis showed that after adjusting other confounding factors, there was still no correlation between having a malignant tumor and the recurrence of atrial fibrillation after radiofrequency ablation (HR = 0.508, 95% CI = 0.192-1.342, P = 0.172).	
Giustozzi et al. (2021) [29]	Retrospective cohort study	Propensity matching of patients with AF and cancer to patients with AF without cancer in a 1:3 and 1:6 ratio after stratification by baseline clinical features.	184	21	NA	30 ± 5 days	The odds ratio (OR) for clinically relevant bleedings was 3.60 (95% CI = 1.02-12.7) higher in cancer than in non-cancer patients.	The lack of data regarding the dose of heparin administered during the procedure is missing in the study to correlate for clinically significant bleeding. There was a lack of generalization, given that it is a single-center retrospective study. The small sample and event size is the drawback of this study.
							In the propensity score-matched population, OR for bleeding was higher in the cancer population (OR = 3.48, 95% CI = 0.76-15.90 for matched 1:3, OR = 4.95, 95% CI = 1.2-20.2 for matched 1:6).	
Voudris et al. (2017) [23]	HCUP-NIS database-based retrospective study	Patients admitted with a diagnosis of atrial fibrillation during the study period with a history of malignancy.	39,486,808	6,718,834	NA	One year	Older people were more likely to have malignancy (77.5 vs. 75.5 years, P < 0.0001).	NA
							All-cause mortality (7.1% vs. 5.2%), length and cost of hospitalization, and blood transfusion rates (15.9% vs. 11.4%) were significantly higher in patients with cancer (P < 0.0001).	
							Rates of permanent pacemaker placement (4.0% vs. 2.5%), DCCV (1.3% vs. 0.9%), and catheter ablation (1.1% vs. 0.5%) were higher in the non-cancer group (P < 0.0001).	
							After multivariate adjustment, independent predictors of mortality included malignancy (OR = 1.39) and acute stroke (OR = 3.80).	

Furthermore, the heterogeneity in study populations, cancer types, type of CA, type of AF, and study designs makes it challenging to draw definitive conclusions. CA for AF is underused in patients with cancer because of the perceived risk of downstream complications [30].

Limitations of the study

The limitations of this study should be acknowledged. Further prospective studies with larger sample sizes are warranted to establish more comprehensive and robust evidence regarding the optimal management strategies and potential risks associated with AF ablation in patients with cancer.

## Conclusions

The major guiding principles for rate or rhythm control in cancer patients are mostly similar to the general population. Specific considerations regarding drug interactions with antiarrhythmic drug therapy, catheter ablation, and electrical cardioversion should be given in cancer patients. Existing evidence from observational studies suggests that catheter ablation for AF in patients with active malignancy or a history of malignancy may have comparable outcomes to that in non-cancer patients. There are limited data, heterogeneity in study populations, and mixed findings regarding procedural bleeding risk to draw a definitive conclusion. Furthermore, the underutilization of catheter ablation in cancer patients highlights the need for further prospective studies with more patients to find out the optimal management strategies and potential risks associated with the procedure.
